# Copper-incorporated bioactive glass-ceramics inducing anti-inflammatory phenotype and regeneration of cartilage/bone interface

**DOI:** 10.7150/thno.36120

**Published:** 2019-08-14

**Authors:** Rongcai Lin, Cuijun Deng, Xuxiang Li, Yaqin Liu, Meng Zhang, Chen Qin, Qingqiang Yao, Liming Wang, Chengtie Wu

**Affiliations:** 1 Department of Orthopaedics, Nanjing First Hospital, Nanjing Medical University. Nanjing 210006, P.R.China; 2 State Key Laboratory of High Performance Ceramics and Superfine Microstructure, Shanghai Institute of Ceramics, Chinese Academy of Sciences. Shanghai 200050, P.R.China; 3 Shanghai East Hospital, the Institute for Biomedical Engineering & Nano Science, Tongji University School of Medicine, 150 Jimo Road, Shanghai 200092, P.R.China

**Keywords:** copper, bioactive glass ceramics, osteoarthritis, cartilage regeneration, immunomodulation

## Abstract

Osteoarthritis not only results in cartilage lesion, but also is accompanied with subchondral bone damage caused by the inflammatory response. It is of great significance to treat osteoarthritis by regulating the immune response. As copper (Cu) plays an essential role in immune response and anti-arthritis, a copper-incorporated bioactive glass-ceramics (Cu-BGC) may achieve the aim of healing cartilage lesion and reducing inflammatory response caused by osteoarthritis. We hypothesized that the Cu^2+^ released from Cu-BGC scaffolds may satisfy the requirements of cartilage regeneration and anti-arthritis.

**Methods**: 3D-printing method was employed to prepare Cu-BGC scaffolds. The stimulating effect on the chondrocytes and macrophages cultured with Cu-BGC extracts was investigated. Furthermore, the *in vivo* regenerative effect of Cu-BGC scaffolds on osteochondral defects was studied.

**Results**: The incorporation of Cu^2+^ into BGC considerably promoted the proliferation and maturation of chondrocytes, and induced macrophages shifting to anti-inflammatory phenotype. Histological analysis demonstrated that the Cu-BGC scaffolds meaningfully improved the regeneration of cartilage and elevated the recovery of the osteochondral interface as compared with the CTR and BGC groups. The potential mechanism is related to Cu^2+^ ions triggering the immune response of cartilage via activating HIF signaling pathway and inhibiting the inflammatory response in osteochondral tissue.

**Conclusion**: These results demonstrated that Cu-BGC scaffolds significantly facilitated the regeneration of cartilage and osteochondral interface, as well as inhibited inflammatory response, which may prevent the development of osteoarthritis associated with osteochondral defects.

## Introduction

Osteoarthritis is a degenerative disease, which is mainly characterized by cartilage wear, degeneration and damage of subchondral bone 1. As articular cartilage has no blood vessels, and subchondral bone has a different physiological property from cartilage. Thus, it is difficult for self-healing of osteochondral tissues 2-4. Previously, chondrocyte transplantation and natural/engineering bone grafts were applied to renew cartilage and subchondral bone in the treatment of osteochondral defects caused by osteoarthritis 4, 5. However, there is no feasible strategy for completely healing of osteochondral defects. In osteoarthritis, pro-inflammatory cytokines promote chondrocytes apoptosis and cartilage matrix proteolysis 6, 7. The inhibition of inflammatory could decrease the damage of cartilage in osteoarthritis 8. Thus, the effects of the immune response in osteoarthritis cannot be ignored. Although there are some studies about immunotherapy of osteoarthritis, there has no study about osteochondral regeneration via activating the immune response of cartilage. Hence, it is of special significance to design a bifunctional material for the regeneration of both articular cartilage and subchondral bone via activating the immune response of cartilage.

Copper is a key trace element, which plays an essential role in maintaining physiological homeostasis and antibiosis in our body 9, 10. Reportedly, copper is important for cellular immunity and humoral immunity 11, 12. Furthermore, the metabolism of copper is obviously enhanced in the acute phase response in inflammation, infection and other diseases 13. As the active site of ceruloplasmin, copper promotes the synthesis and secretion of ceruloplasmin via stimulating the synthesis of inflammatory cytokines and hypoxia-inducible factor 14-16.

In healthy tissues especially cartilage, copper is an important component in the synthesis of cellular enzymes, such as copper-containing superoxide dismutase, cytochrome oxidase and lysyl oxidase 17. Particularly, lysyl oxidase, a copper-dependent amine oxidase, has been proved to be a key enzyme for collagen cross-linking and further promotes the formation of cartilage 18, 19. Furthermore, copper deficiency could decrease bone strength and increase the incidence of osteoarthritis 20. In addition, copper was demonstrated has a positive effect on osteogenesis and anti-arthritis 21, 22. Based on the osteoconductivity of bioactive glass ceramics (BGC) 23, 24, the incorporation of copper into BGC was supposed to have the capability to stimulate osteochondral regeneration and anti-inflammation via activating the immune response of cartilage. Herein, a copper-incorporated BGC (Cu-BGC) scaffold was developed, and the *in vitro* and *in vivo* bioactivities as well as the underlying mechanisms of cartilage regeneration and immune response were systematically investigated.

## Methods

### Cu-BGC powders preparation and characterization

BGC and Cu (5 mol%)-doped BGC were prepared by a sol-gel method according to our previous publication 25. First, 25 mL of nitric acid (HNO_3_, 2 M) and 157 mL of Tetraethyl orthosilicate ((C_2_H_5_O)_4_Si, TEOS) were dropwise added into 100 mL of ultrapure water, and the mixture was stirred continuously for half an hour to obtain a TEOS solution. Subsequently, 12.08 g of copper nitrate trihydrate (Cu(NO_3_)_2_**·**3H_2_O), 47.23 g of calcium nitrate tetrahydrate (Ca(NO_3_)_2_**·**4H_2_O) and 8.53 mL of triethyl phosphate ((C_2_H_5_O)_3_PO) were mixed with the aforementioned TEOS solution (the above chemical raw materials were obtained from the Sinopharm Chemical Reagent Co., Ltd). After stirring for 5 h, in order to obtain wet gels, the solution was kept static for 48 h at 60℃, followed by drying the wet at 120℃ for 24 h to obtain a dry product. After grinding, the Cu-BGC powders were sintered at 800℃ (2℃ min^-1^). The pure BGC, which acts as a control, was prepared through a similar way.

### Cu-BGC scaffolds preparation and characterization

In this study, Auto CAD was employed to design the mesh-like structure of Cu-BGC scaffolds and 3D printing method was used to fabricate the scaffolds. 3D-printing ink used for fabricating Cu-BGC scaffolds was obtained by mixing of Cu-BGC powders (5.00 g), sodium alginate (0.15 g) with 20wt% F-127 solution (3.5 g). Then the printable glass ceramic ink was loaded, and printed through a 22G needle (inner diameter was 0.22 mm) to obtain the primary scaffold. The dosing pressure was 3.0-6.0 bar and the moving speed of the needle was 6 mm s^-1^. In order to obtain the final products, primary products were sintered at 1300 °C (2℃ min^-1^) for 3 h after drying at room temperature. As a control, BGC scaffolds were prepared through a similar way. Macroscopic photograph and surface morphology of Cu-BGC and BGC scaffolds were characterized by a Nikon digital camera and a HITACHI scanning electron microscope (SEM) from Japan, respectively.

The compressive strengths of BGC and Cu-BGC scaffolds were tested by a computer-controlled universal testing machine (AG-I, Shimadzu, Japan) with a speed of 1.0 mm/min. In order to evaluate the degradation behavior of Cu-BGC scaffolds and assay Cu^2+^ ions release from Cu-BGC scaffolds, the scaffolds were soaked in Tris-HCl solution at a ratio of 200 mL/g (Tris-HCl volume/scaffolds mass) in the test tubes (n=3), then these tubes were placed in a shaking water bath at 37 °C for 1, 3, 7, 14, 21, 28 d, respectively. After the set soaking time, the scaffolds were removed from Tris-HCl solution and dried in an air oven at 60 °C for 24 h, and the weight of each scaffold was measured. The weight loss of the scaffolds was expressed by the ratio of the final weight to the initial weight. The Tris-HCl was replaced and collected at each time point. The concentrations released of Cu^2+^ in solutions were detected by inductively coupled plasma atomic emission spectrometry technique (ICP-AES; Varian, US).

### Cell culture of chondrocytes and macrophages

Chondrocytes (New Zealand white rabbit) and murine-derived macrophage cell line RAW264.7 (RAW) cells were used in this study. The rabbit chondrocytes were provided by Nanjing Medical University Nanjing Hospital. The chondrocytes were freshly isolated by experienced technicians from New Zealand white rabbits, and the application of the chondrocytes for in vitro study was approved by the ethics commission Nanjing Medical University. The RAW264.7 cells were purchased from Cyagen Biosciences (China). A Dulbecco's Modified Eagle's Medium low-glucose (DMEM, Life Technologies, America) with 1% (v/v) penicillin/streptomycin (Thermo, America) and 10% fetal calf serum (Thermo, America) was used for culturing chondrocytes. The DMEM high-glucose (Life Technologies, USA) contained 1% (v/v) penicillin/streptomycin (Thermo, America) and 10% fetal bovine serum (Thermo) was used for RAW cells culturing. Chondrocytes and RAW cells were incubated in a 37°C incubator with 5% CO_2_.

### The proliferation and differentiation of chondrocytes incubated with the ionic products of Cu-BGC

The proliferation of chondrocytes incubated with the dilute ionic products from Cu-BGC and BGC was measured by a cell counting assay (CCK-8 kit, Beyotime, China). In brief, the chondrocytes were cultured in 96-well plates for 1, 3, 5, 7 days with a primary density of 1500 cell/well. Afterwards, cells were incubated with a 10% CCK-8 culture medium for one and half hours in a cell culture incubator. Absorbance values of culture medium were investigated by utilizing a Spectra Fluor Plus microplate reader (Germany) at 450 nm wavelength.

BGC and Cu-BGC extracts were prepared according to the following protocol: the powders of BGC or Cu-BGC were dissolved in DMEM at a standard ratio of 200 mg/mL (DMEM volume/powder mass), and vibrated in a 37 °C shaker (120 r/min) for 24 h. Then the mixtures were centrifuged (4000 rpm/min, 10 min) and filtrated (0.22 µm filters). Finally, the raw extracts of BGC and Cu-BGC were diluted into 100 mg/mL, 50 mg/mL, 25mg/mL, 12.5 mg/mL, 6.25 mg/mL, 3.125 mg/mL, 1.5625 mg/mL and 0.78125 mg/mL, respectively.

Quantitative real-time reverse transcriptase polymerase chain reaction (RT-qPCR) was conducted for investigating the maturation of chondrocytes. In brief, the chondrocytes were inoculated into a 6-well plate with dilute extracts (25 mg/mL, 12.5 mg/mL, 3.125 mg/mL and 0.781 mg/ mL) of BGC and Cu-BGC for 3 days. Then the chondrocytes were processed the treatment of a TOYOBO Micro Kit (Japan) to obtain total RNA. The total RNA concentration was detected by a Spectra Fluor Plus microplate reader (Germany) at 260 nm wavelength. To prepare cDNA, a TOYOBO cDNA synthesis kit was applicated. The RT-qPCR process was conducted in a Bio-rad Light Cycler apparatus (CFX Touch) with a TOYOBO SYBR Green qPCR Kit. Finally, a 2^-ΔΔCt^ method was used to calculate the expression level of target genes. Besides, to study the potential mechanism of the ionic extracts supported chondrocytes differentiation, HIF-1α gene was examined. All of the primer sequences of target genes were developed via oligo 7.0 software and shown in Table S1.

### The expressions of NCAD and type II collagen proteins in chondrocytes cultured with Cu-BGC extracts

An Abcam type II collagen protein staining kit (America) and an Abcam N-cadherin (NCAD) protein staining kit (America) were employed to evaluate type II collagen and NCAD protein expressed in chondrocytes. Firstly, chondrocytes were cultured with the ionic extracts of BGC and Cu-BGC for 3 days, and then fixed with 2.5% gluteraldehde (Sinopharm Group Co., Ltd). And then the chondrocytes were incubated for 1 h by using 1% bovine testicular hyaluronidase. Afterwards, the primary antibody (COL II, Abcam: ab3092; NCAD, Abcam: ab19348, respectively) was used to incubate the chondrocytes for 12 h at 4°C, and second antibody (COL II: Abcam: ab150105; NCAD, Abcam: ab150107, respectively) was applied to incubate the cells at 37°C for 1 h. Finally, the cell cytoskeleton was stained by a fluorescein isothiocyanatephalloidin (FITC, Sigma-Aldrich, America) solution, and the nuclei were stained by a 4', 6-diamidino-2-phenylindole (DAPI, Sigma-Aldrich, America) solution. Fluorescent photographs were captured by a confocal laser scanning microscopy (CLSM, Leica TCS SP8). The contents of proteins were analyzed using Image pro-plus 6.0 (Media Cybernetics, US) software.

### The proliferation and inflammatory response of macrophages cultured with the extracts of Cu-BGC

The proliferation of macrophages (RAW cells) was investigated via a CCK8 kit (Beyotime, China) as we described before. Then, RT-qPCR was conducted to analyze the expression of inflammatory cytokine genes (TNF-α, IL-18, IL-10) and macrophage-phenotype surface marker genes (iNOS, CD206). RAW cells were cultured with dilute solutions (25 mg/mL, 12.5 mg/mL, 3.125 mg/mL and 0.781 mg/ mL) of BGC and Cu-BGC for 1 and 3 days. Afterwards, the expressions of inflammatory genes were obtained by using the protocol we described before. All of the primer sequences of target genes were shown in Table S1.

### The stimulating effects of Cu^2+^ ions on proliferation and differentiation of chondrocytes and macrophages

The gradient Cu^2+^ ions solutions were prepared by the way we described before through using CuCl_2_ salt. The proliferation of macrophages and chondrocytes cultivated with distinct concentrations of Cu^2+^ ions (the concentrations are 128, 64, 32, 16, 8, 4, 2, 1, 0.5 ppm for Cu^2+^, respectively) was investigated by the same method we described before. Furthermore, total RNA was collected after the chondrocytes were incubated with Cu^2+^ ions for 3 days, and the expression levels of target genes (COL II, aggrecan (ACAN), SOX-9 and HIF-1α) were measured. CLSM was used to investigate the secretion of target proteins (ACAN, NCAD and COL II) by chondrocytes treated with different concentrations of Cu ions (16, 8, 2 and 0.5 ppm, respectively) at day 3.

Moreover, a similar way was employed to investigate the proliferation and inflammatory genes expressions of the macrophages cultured with different concentrations of Cu^2+^ ions.

### The attachment of chondrocytes on Cu-BGC scaffolds *in vitro*

To observe the adhesion of chondrocytes in BGC and Cu-BGC scaffolds, the chondrocytes were seeded in BGC and Cu-BGC scaffolds for 12 h. Subsequently, the cellular samples were treated with glutaraldehyde (2.5%) and graded ethanol (30%, 50%, 70%, 80%, 90%, 95%, and 100%). The morphological characteristic of chondrocytes on scaffolds was observed by using a HITACHI SEM (Japan). Besides, the cytoskeleton and nuclei of chondrocytes were stained by FITC and DAPI to observe CLSM images, respectively.

### The regeneration of cartilage and subchondral bone for Cu-BGC scaffolds i*n vivo*

The Ethics Committee of Nanjing First Hospital, Nanjing Medical University approved the protocol of animal experiment. The rabbit model for osteochondral defects has been widely applied. Twenty-four 3 months old New Zealand white rabbits (2-2.5 kg) were used to build osteochondral defect models for assessing the reconstruct effect of BGC and Cu-BGC scaffolds. Previous studies showed that the critical size of osteochondral defect for rabbits to spontaneous healing is 3 mm 26, 27. It is difficult for rabbit to spontaneous healing osteochondral defects large than 3mm. In this study, osteochondral defects with a diameter of 5 mm was employed to investigate the *in vivo* regenerate effect. The osteochondral defects (height: 5mm, diameter: 5 mm) were fabricated on the femoral condyle after general anesthesia, and then BGC and Cu-BGC scaffolds (height: 5mm, diameter: 5 mm) were transplanted into the defect sites (Figure S6). The defects regions of blank control group had no scaffold implanting. In BGC group and Cu-BGC group, BGC scaffolds and Cu-BGC scaffolds were implanted, respectively. In the above experimental groups, there were six parallel samples in each group. All rabbits were injected with antibiotics for three days. After 8/12 weeks post operation, New Zealand white rabbits were sacrificed and the condyles of femur were collected for investigation of regeneration effect by using the digital camera, Micro-CT and histological analysis. The Micro-CT images were obtained from a Micro-CT scanner (SKYSCAN 1172, BRUKER, Germany). The scanning was performed under the maximum voltage of 80 kV and maximum beam of 100 μA. The knee samples were placed in a scanning tube after soaking in saline for 24 h. Then the samples were scanning under the consideration of 8.9 μm resolution, 80 kV voltage and aluminum & copper filter. Subsequently, the obtained data was analyzed within a thresholding range of 50-255. Finally, a CT vox-short cut software was used to obtain the final images. The international cartilage repair society macroscopic assessment (ICRS) was used to assess the regeneration of cartilage. And the relative bone volume fraction (BV/TV) was obtained from the micro-CT. Digital camera was used to observe the gross morphology of samples. Besides, Van Gieson staining (VG) and Hematoxylin-eosin (HE) staining were used to assess the repair situation of cartilage and subchondral bone tissues regeneration.

### Statistical analysis

All the data were expressed as means ± standard deviation and the significance of experimental data were analyzed using T-Test and ANOVA followed by post hoc test. *P < 0.05, **P <0.01, ***P < 0.001.

## Results

### *In vitro* stimulatory effects of Cu-BGC ionic extracts on proliferation and maturation of chondrocytes

**Figure 1:**
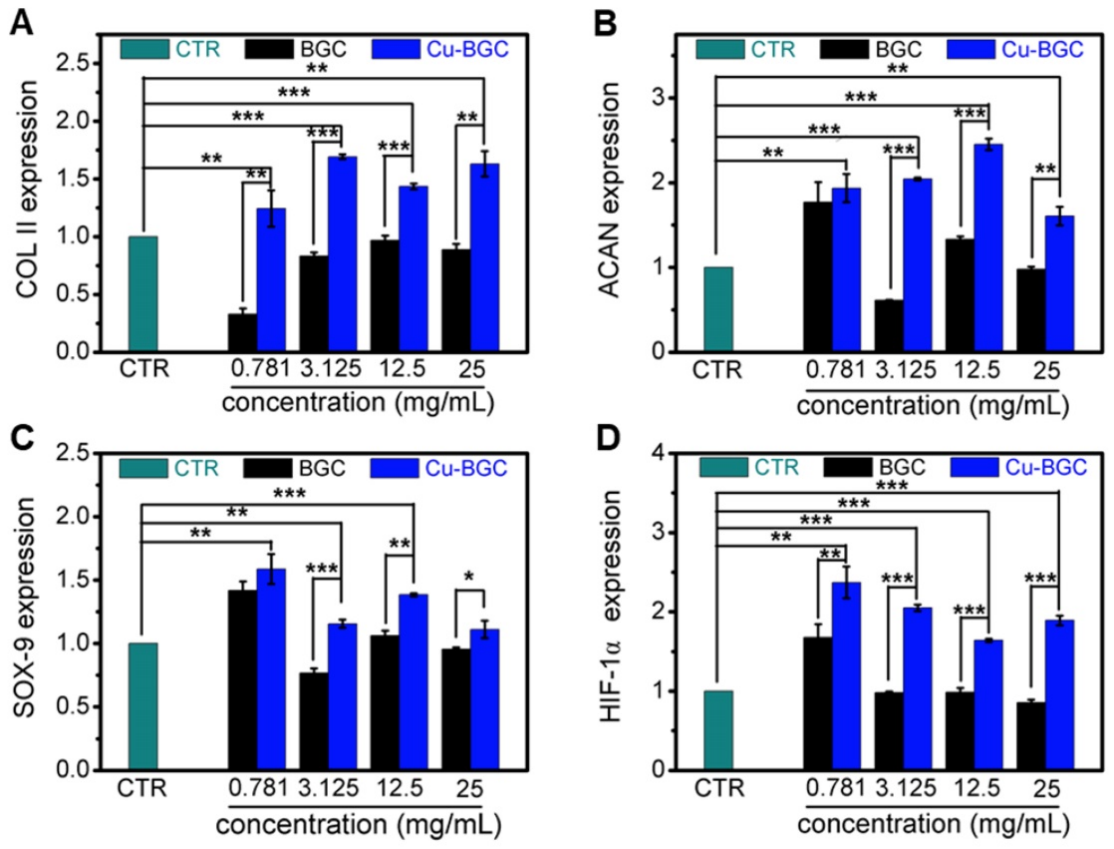
The relative genes expression in chondrocytes cultured with the ionic extracts of BGC and Cu-BGC for 3 days. (A) COL II, (B) ACAN, (C) SOX-9, (D) HIF-1α. The ionic extracts of Cu-BGC significantly enhanced the chondrogenic differentiation of relative genes of chondrocytes within the concentration range of 3.125-25 mg/mL. The relative gene amount of CTR group was set as 1. (n=3, *p<0.05, **p<0.01, ***p<0.001)

**Figure 2:**
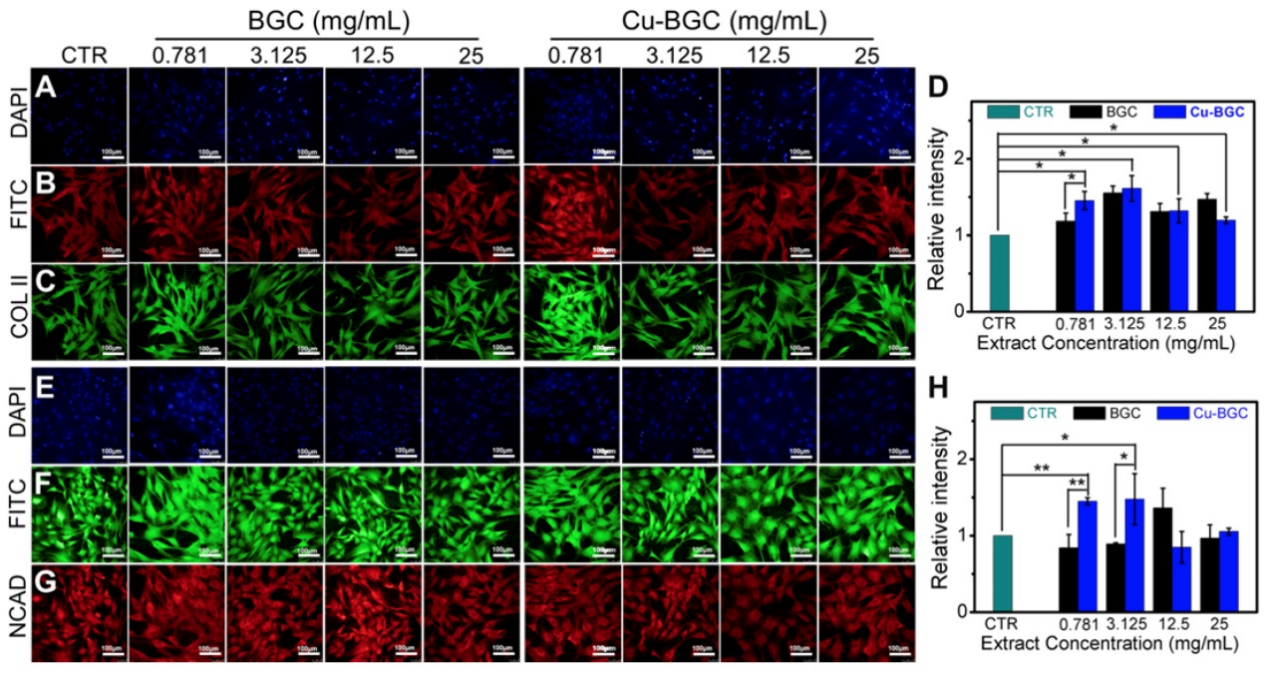
The COL II, NCAD proteins distributed in chondrocytes cultured with the ionic extracts of BGC and Cu-BGC for 3 days. (A, E) DAPI, (B, F) FITC, (C) COL II, (G) NCAD. The corresponding intensity of COL II (D) and NCAD (H). As compared with BGC group, the ionic extracts from Cu-BGC increased the expression of COL II and NCAD proteins in chondrocytes within certain concentration ranges. (scale bar=100 μm, n=6, *p<0.05, **p<0.01, ***p<0.001)

As compared with BGC and CTR (blank control) groups, the proliferation of chondrocytes treated with Cu-BGC ionic extracts (0.781-3.125 mg/mL) was promoted (Figure S1A). The results of RT-qPCR analysis revealed that the extracts of Cu-BGC significantly enhanced the chondrocytes specific genes (COL II, SOX-9, ACAN) expression as compared with CTR and BGC groups within a certain concentration range (3.125-25 mg/mL) at 3 days (Figure 1A-C). In order to examine the potential mechanism of Cu^2+^ ions promoting the proliferation and maturation of chondrocytes, the HIF pathway was detected by using RT-qPCR analysis. Results revealed that the expression level of HIF-1α was distinctly enhanced after incubated with Cu-BGC ionic extracts for 3 days as compared with BGC and CTR groups within a concentration range of 0.781-25 mg/mL (Figure 1D). Furthermore, the expression level of type II collagen protein (Figure 2A-D) and NCAD protein (Figure 2E-H) in chondrocytes treated with Cu-BGC extracts was improved within the certain concentration ranges.

### The stimulating effects of Cu^2+^ ions on the proliferation and maturation of chondrocytes

To investigate the effect of Cu^2+^ ions on the proliferation and maturation of chondrocytes, the cells were incubated with different Cu^2+^ ions concentrations. The CCK-8 results displayed that the proliferation capability of chondrocytes treated with different Cu^2+^ ions concentrations increased within the concentration range of 0.5-2 ppm (Figure S1B).

**Figure 3:**
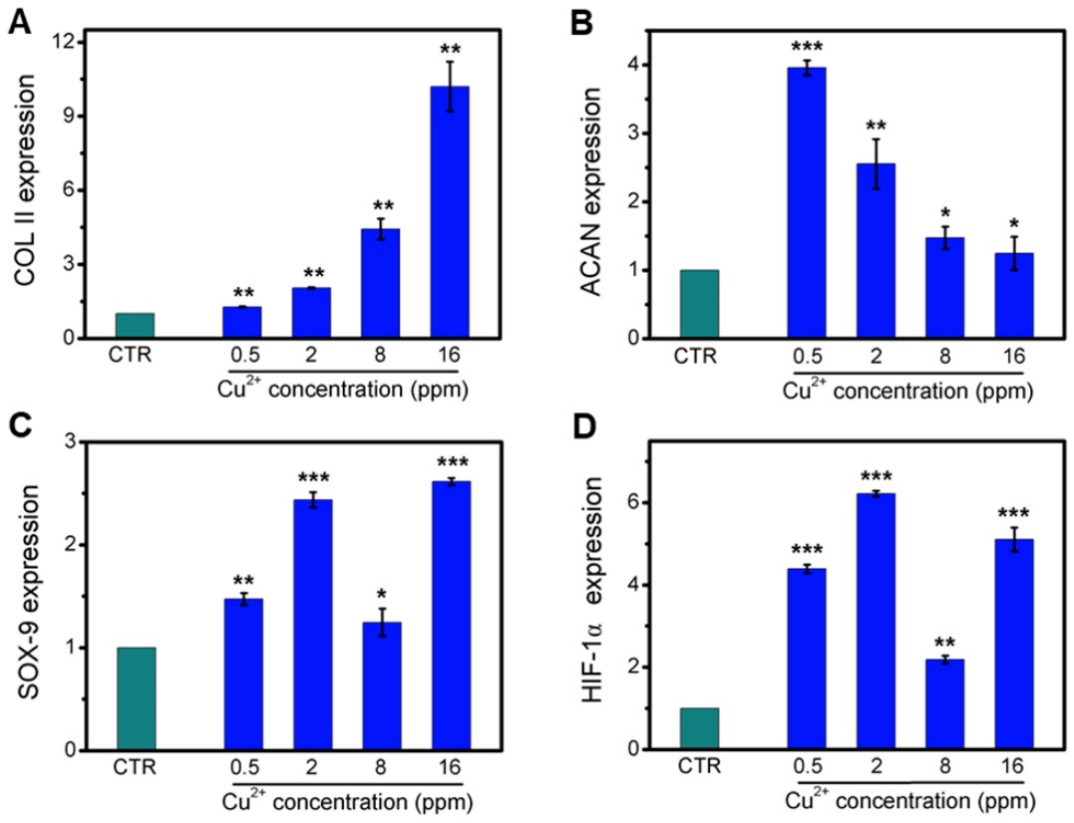
The relative genes expression in chondrocytes treated with different concentrations of Cu^2+^ ions for 3 days. (A) COL II, (B) ACAN, (C) SOX-9, (D) HIF-1α. After treating with different concentrations of Cu^2+^ ions, the relative genes of chondrocytes were significantly enhanced. The relative gene amount of CTR group was set as 1. (n=3, *p<0.05, **p<0.01, ***p<0.001)

**Figure 4:**
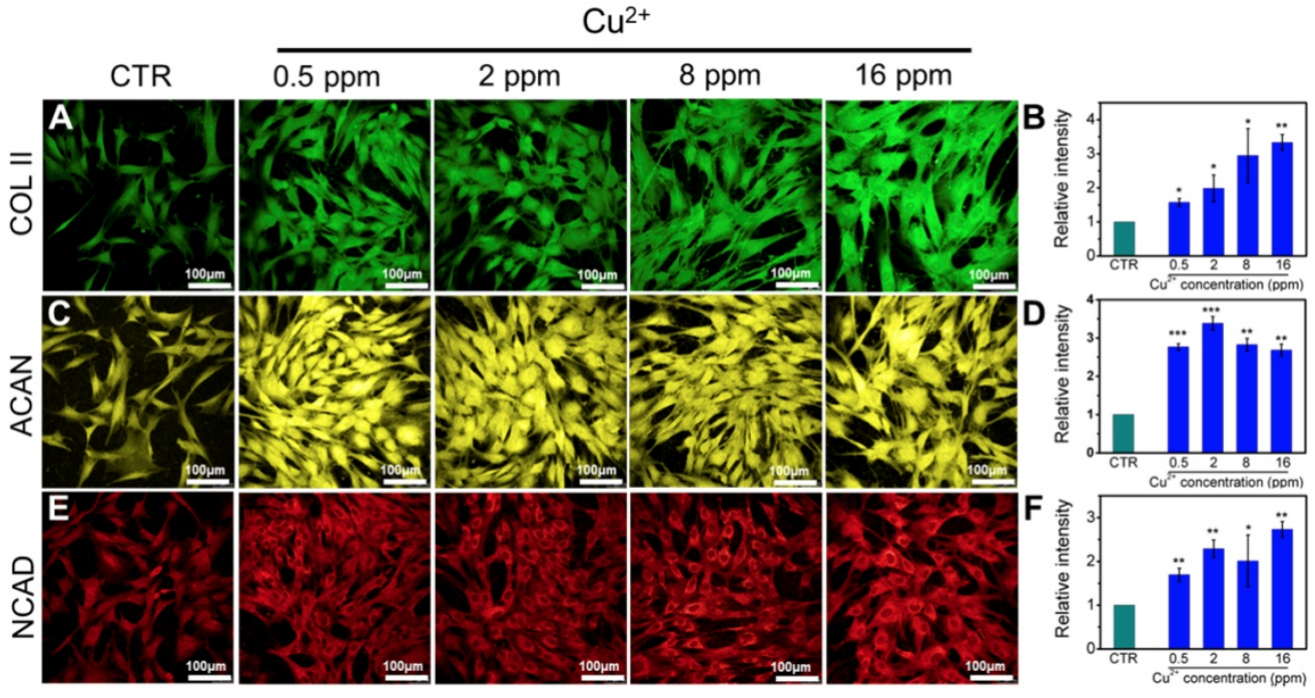
The expression of COL II, ACAN, NCAD proteins in chondrocytes cultured with different concentrations of Cu^2+^ ions for 3 days. (A) COL II, (C) ACAN, (E) NCAD. The corresponding intensity of COL II (B), ACAN (D) and NCAD (F). The different concentrations of Cu^2+^ ions enhanced the expression of COL II, ACAN and NCAD in chondrocytes as compared with CTR group. (Scale bar = 100 μm, n=6, *p<0.05, **p<0.01, ***p<0.001)

Collagen X is a representative gene of hypertrophic chondrocytes, while SOX-9 is a positive factor for inhibiting hypertrophic differentiation of chondrocytes. In this study, the expression of SOX9 was mainly investigated, and results indicated that the expression of SOX9 was significantly upregulated after treating with Cu^2+^ ions. Furthermore, other target genes (COL II and ACAN) expression in chondrocytes was significantly enhanced after cultured with different Cu^2+^ ions concentrations within a concentration range of 0.5-16 ppm at day 3 (Figure 3A-C). In particular, Cu^2+^ ions obviously promoted HIF-1α gene expression in chondrocytes as compared with CTR group (Figure 3D). Additionally, the expression of NCAD protein and COL II protein was further investigated. The *in vitro* results showed that Cu^2+^ ions markedly elevated expressions of COL II protein (Figure 4A, B) and NCAD protein (Figure 4E, F) within the concentration of 0.5-16 ppm as compared with CTR groups at day 3.

### Cu-BGC ionic extracts inducing macrophages to M2 phenotype

**Figure 5:**
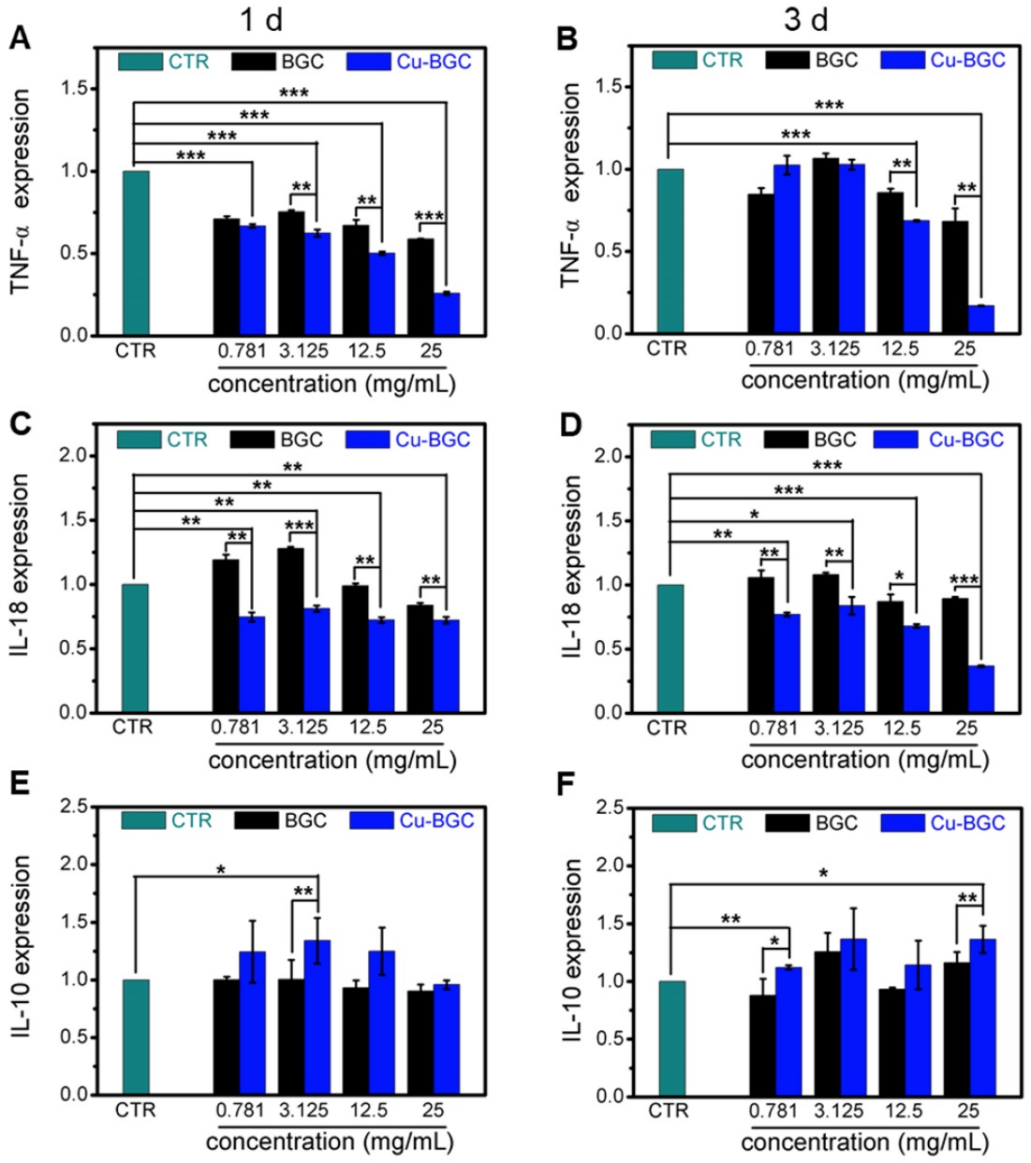
The inflammatory cytokine expression in macrophages cultured with BGC and Cu-BGC ionic products. The expression of TNF-α genes (A, B), IL-18 genes (C, D) were downregulated by the stimulation of Cu-BGC extract at day 1 and 3, while IL-10 genes (E, F) were upregulated. The results indicated that Cu-BGC ionic products inhibited pro-inflammatory cytokine expression, and elevated anti-inflammatory cytokine expression. The relative gene amount of CTR group was set as 1. (n=3, *p<0.05, **p<0.01, ***p<0.001)

**Figure 6:**
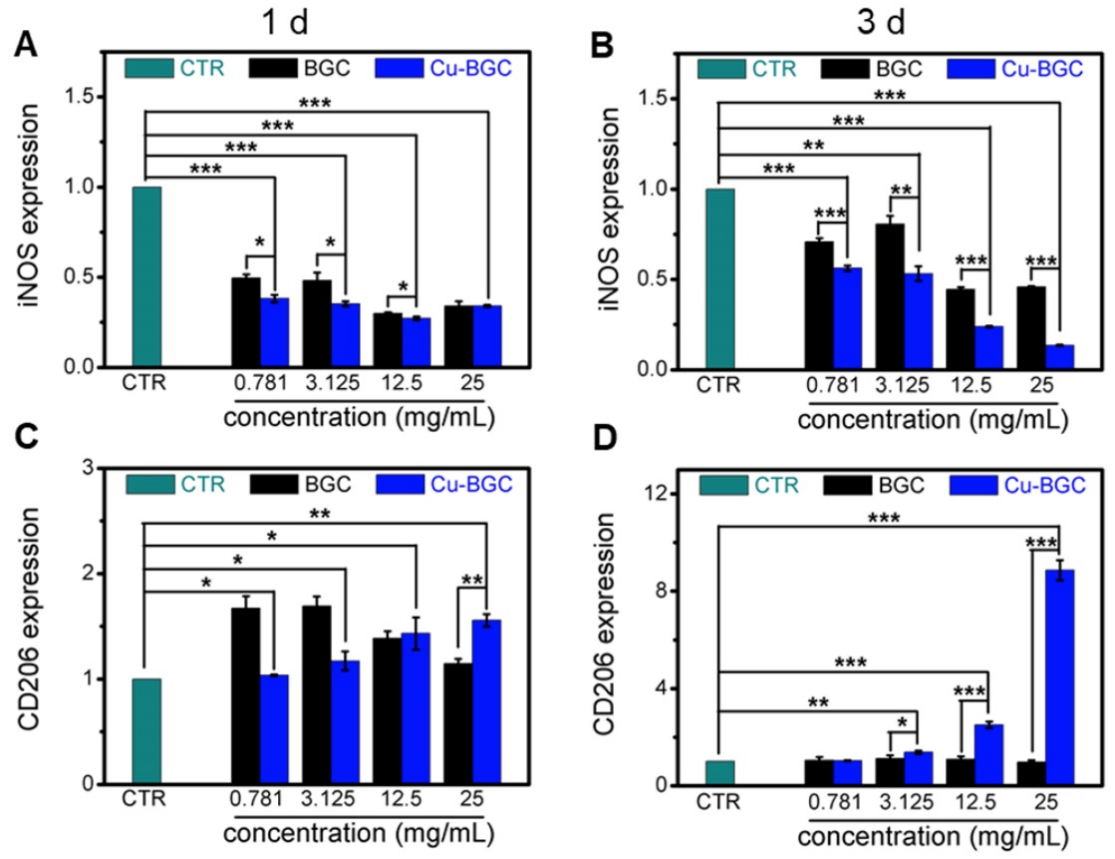
The expression of macrophage surface markers incubated with BGC and Cu-BGC ionic products at day 1 and 3.(A, B) M1 marker: iNOS; (C, D) M2 marker: CD206. It was found that the Cu-BGC ionic products induced the macrophages polarizing to an anti-inflammatory M2 phenotype and inhibited the macrophages shifted to a pro-inflammatory M1 phenotype. The relative gene amount of CTR group was set as 1. (n=3, *p<0.05, **p<0.01, ***p<0.001)

The results of CCK-8 indicated that the ionic extracts from Cu-BGC have the possibility to promote the proliferation of RAW cells compared with CTR group within the concentration range of 0.781-3.125 mg/mL at 3 days, while there was no obvious difference from CTR group at day 1 and 2 (Figure S2A). To assess the effects of Cu-BGC extracts on macrophages, the expressions of inflammatory cytokine and macrophages surface markers were examined. The gene expression of the pro-inflammatory cytokine (TNF-α, IL-18) in macrophages cultured with Cu-BGC ionic extracts was inhibited as compared with BGC and CTR groups within a concentration of 0.781-25 mg/mL at day 1 and 3 (Figure 5A-D). While the anti-inflammatory cytokine gene expression (IL-10) was improved in macrophages after culturing with Cu-BGC extracts as compared with BGC and CTR group within the certain concentration ranges (Figure 5E, F). Furthermore, the expression of M1 surface marker (iNOS) in macrophages treated with Cu-BGC extracts was obviously down-regulated as compared with BGC and CTR groups within a concentration range of 0.781-25 mg/mL at 1 and 3 days (Figure 6A, B). While M2 surface marker (CD206) expression was obviously enhanced after co-culturing with Cu-BGC extracts for 3 days as compared with BGC and CTR groups within the certain concentration ranges. Particularly, the expression of CD206 in macrophages cultured with Cu-BGC extracts was distinctly upregulated at the concentration of 25 mg/mL (Figure 6C, D). These results demonstrated that the ionic extracts from Cu-BGC have the capability to facilitate the macrophages from M0 phenotype to an anti-inflammatory M2 phenotype.

### The polarization effects of Cu^2+^ ions on macrophages

According to the concentrations of Cu^2+^ ions in Cu-BGC extracts, the macrophages response to the different concentrations of Cu^2+^ ions was investigated. In CCK-8 analysis, Cu^2+^ ions enhanced the proliferation of macrophages as compared with CTR group within the concentration range of 0.5-4 ppm (Figure S2B). In RT-qPCR analysis, the expression of pro-inflammatory cytokine (TNF-α) in macrophages cultured with different concentrations of Cu^2+^ ions was suppressed as compared with CTR group at day 1 and 3 within certain concentration range (Figure S3A, B). Cu^2+^ ions improved the pro-inflammatory cytokine (IL-18) expression at day 1, while significantly inhibited the expression of IL-18 at day 3 within concentration of 0.5-16 ppm as compared with the CTR group (Figure S3C, D). The expression of anti-inflammatory cytokine (IL-10) was elevated as compared with the CTR group (Figure S3E, F). Moreover, the expression of M1 surface marker (iNOS) and M2 surface marker (CD206) in macrophages cultured with different concentrations of Cu^2+^ ions was examined, and the results showed that iNOS expression was down-regulated and CD206 expression was upregulated at day 1 and 3 within a concentration of 0.5-16 ppm (Figure S4).

### The micromorphology and adhesion of chondrocytes in Cu-BGC scaffolds

**Figure 7:**
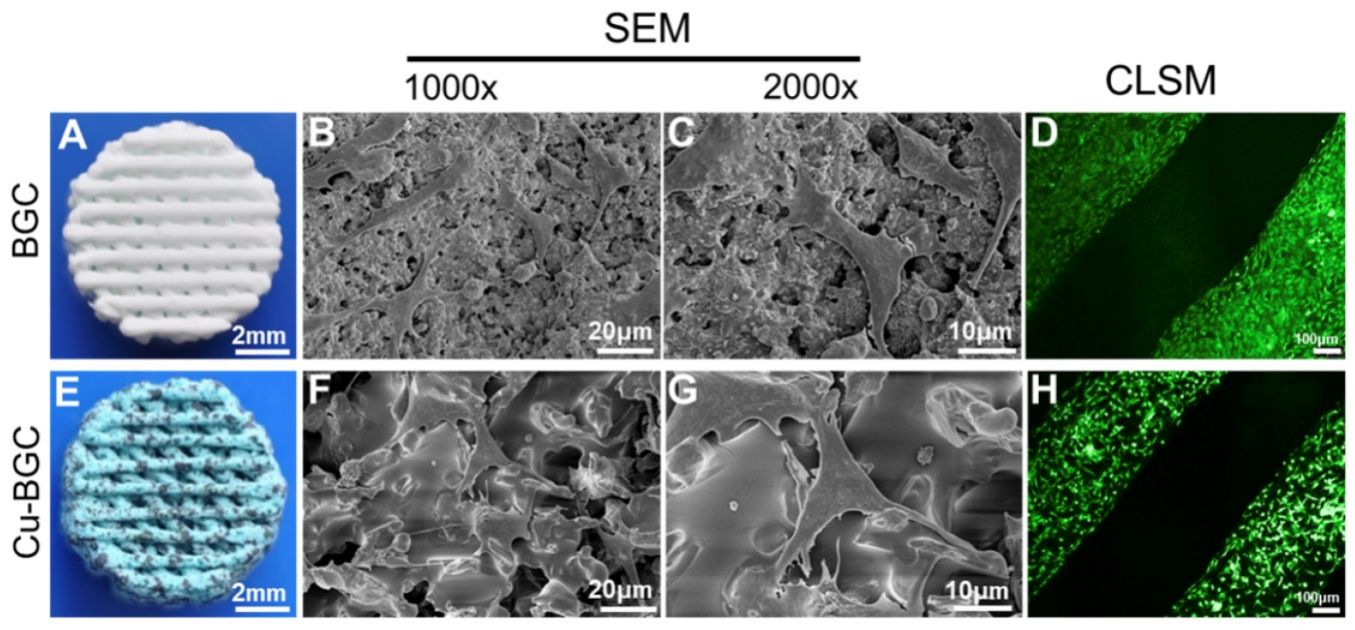
The structure of scaffolds and the morphology of chondrocytes cultured in BGC and Cu-BGC scaffolds. The gross morphology of BGC (A) and Cu-BGC (E) scaffolds. The SEM images of BGC (B) and Cu-BGC (F) scaffolds. The SEM images of chondrocytes after seeding in BGC (C) and Cu-BGC (G) scaffolds, and CLSM images of chondrocytes after seeding in BGC (D) and Cu-BGC (H) scaffolds. As compared to BGC scaffolds, the chondrocytes in Cu-BGC scaffolds showed better-defined microfilaments.

The surface structure of 3D-printed scaffolds and morphology of chondrocytes in scaffolds were shown in Figure 7. The overall morphology of scaffolds was displayed in Figure 7A and Figure 7E. It was found that scaffold showed a blue color after incorporating copper. Furthermore, BGC and Cu-BGC scaffolds exhibited a mesh-like structure. SEM images exhibited that the surface of Cu-BGC scaffold was much denser than BGC scaffold after incorporating copper (Figure 7B, Figure 7F). After chondrocytes were seeded in BGC and Cu-BGC scaffolds, SEM and CLSM were used to investigate the morphology of cells on the scaffolds. SEM images demonstrated that chondrocytes in BGC and Cu-BGC scaffolds spread well and have rich pseudopodia (Figure 7C, Figure 7G). Furthermore, CLSM images further confirmed that BCG scaffold and Cu-BGC scaffold have the capability to support chondrocytes adhesion (Figure 7D, Figure 7H).

### The characterization of Cu-BGC scaffolds

**Figure 8:**
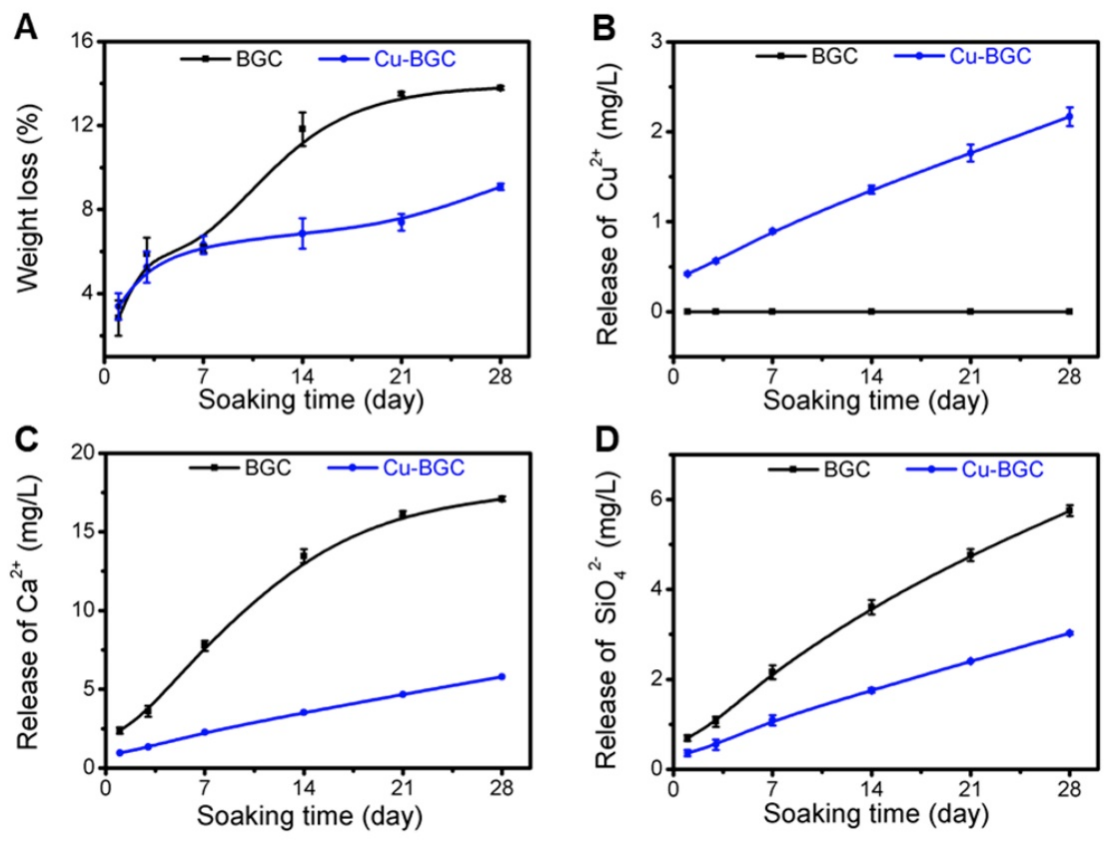
Characterization of BGC and Cu-BGC scaffolds. (A) Weight loss of BGC and Cu-BGC scaffolds in Tris-HCl solution (pH=7.4); (B-D) the accumulative release profiles of Cu^2+^, Ca^2+^, SiO_4_^2-^in Tris-HCl solution. It was found that Cu-BGC scaffolds maintained a sustained release of Cu^2+^ ions. (n=3)

The compressive strength of Cu-BGC scaffold was higher than that of BGC scaffold (Figure S5). The degradation performance of BGC and Cu-BGC scaffolds maintained in Tris-HCl solution for 28 days was 13.78% and 9.08%, respectively (Figure 8A). Furthermore, it was found that Cu-BGC scaffolds maintained a sustained release of Cu^2+^, Ca^2+^ and SiO_4_^2-^ ions (Figure 8B-D).

### Cu-BGC scaffolds stimulating the *in vivo* osteochondral regeneration

**Figure 9:**
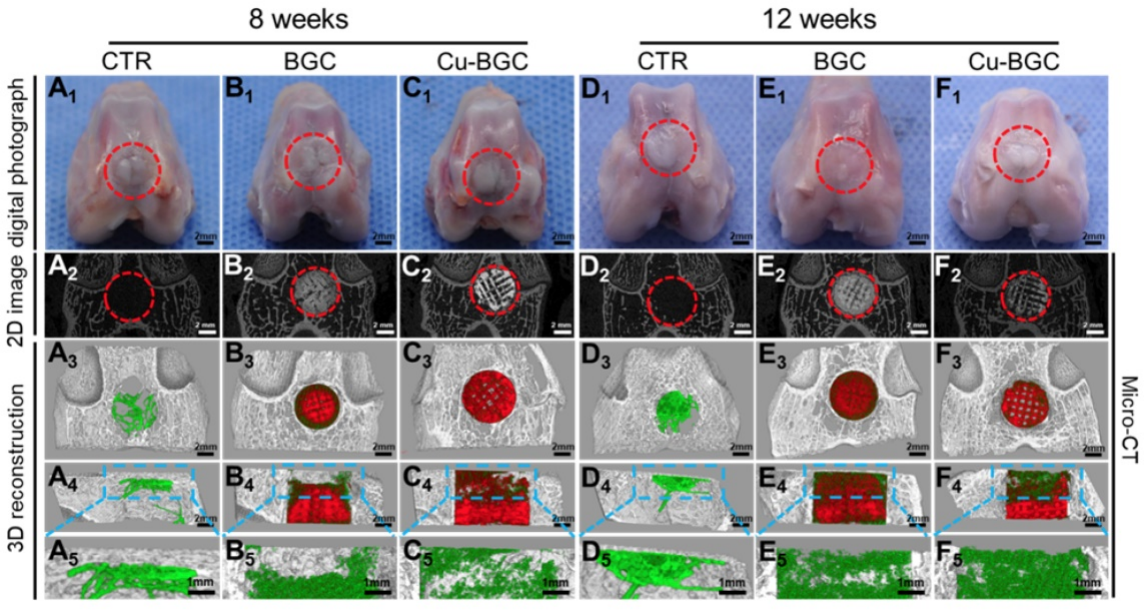
The gross morphology and Micro-CT images of the samples at 8 and 12 weeks of post-surgery. (A_1_-F_1_) The digital photographs of the samples, (A_2_-F_2_) 2D projection images of the defects, (A_3_-F_3_) and (A_4_-F_4_) showed the transverse view and sagittal view of 3D construction image, respectively. (A_5_-F_5_) The images of new bone in the upper part of the defects. In 3D reconstruction images, the off-white color, green color and red color stand for primary bone, new bone and scaffolds, respectively. As compared with CTR and BGC groups, Cu-BGC group displayed a considerable amount of neo-bone tissue in the defect region at 12 weeks.

Previously, the rabbit model for osteochondral defects has been widely applied, and the critical size of osteochondral defect for rabbits to spontaneous healing is 3 mm 26, 27. Hence, the efficacy of Cu-BGC scaffolds for *in vivo* osteochondral regeneration was assessed in a femoral condyle osteochondral defect model (height: 5mm, diameter: 5 mm), which created in New Zealand White rabbits. Gross morphology and Micro-CT analysis of animal samples collected at week 8 and 12 were displayed in Figure 9. Gross morphology of knee samples displayed that there was no obvious inflammatory response observed, and neo-tissue was filled with the defect region in BGC and Cu-BGC groups at 12 weeks (Figure 9A_1_-F_1_). The defects in Cu-BGC and BGC groups were filled with much more calcified tissue than that in the CTR group at week 8 and 12 (Figure 9A_2_-F_2_). Compared to CTR and BGC groups, Cu-BGC group exhibited a greater amount of green color stained tissue within the defect region in 3D reconstruction images at week 12 (Figure 9F_3_-F_5_), which demonstrate that Cu-BGC have a superior performance for bone formation. As compared with CTR group, the Micro-CT analysis displayed that the defects of BGC and Cu-BGC groups existed much more calcified tissue (Figure 9A_2_-F_5_). Furthermore, the relative bone volume fraction (BV/TV) in the defect of Cu-BGC group was higher than the CTR group at week 12 (Figure S7).

**Figure 10:**
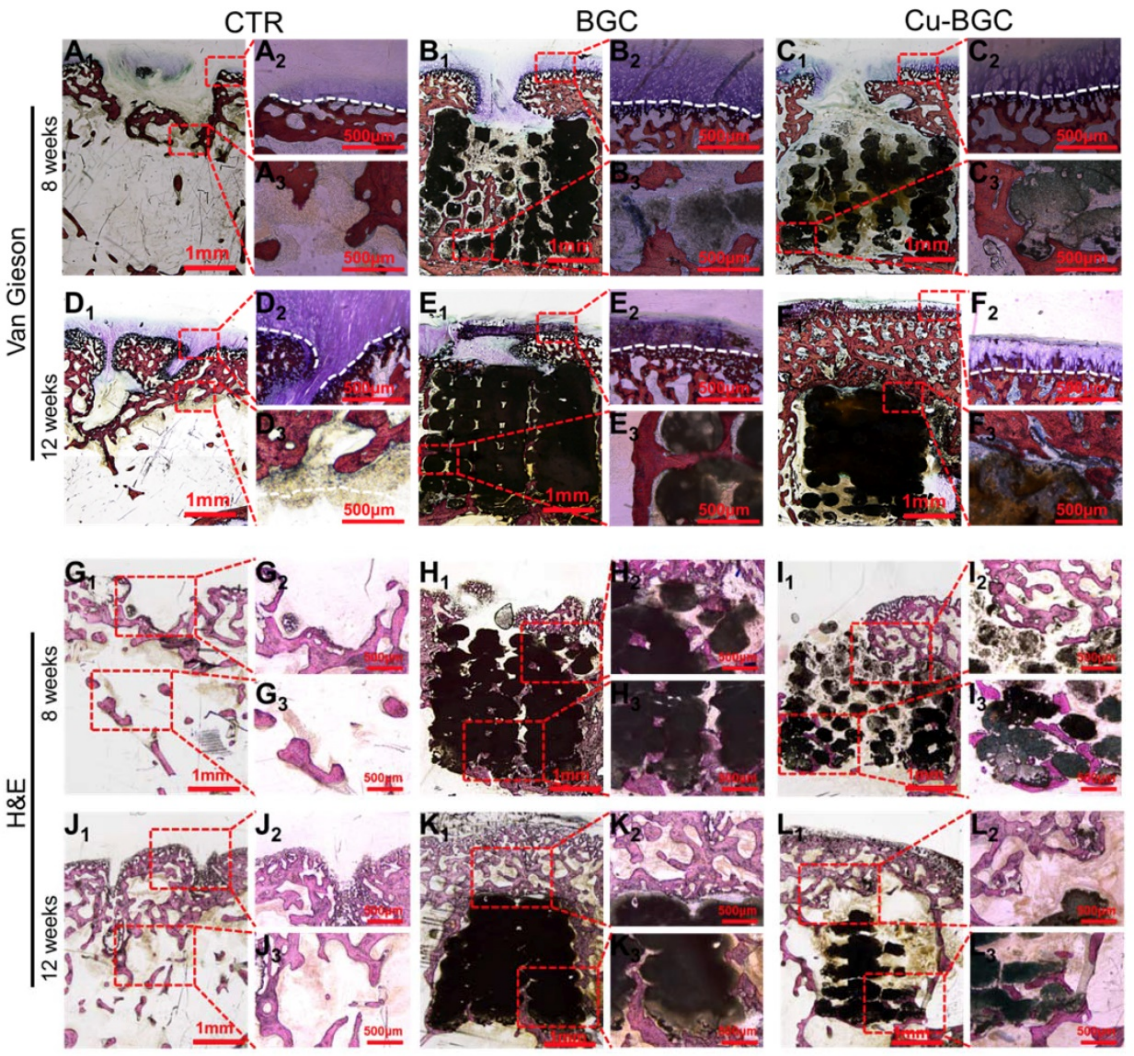
The cartilage and subchondral bone regeneration *in vivo* at 8 and 12 weeks of post-surgery. (A_1_-F_3_) Van Gieson staining at 8 (A_1_-C_3_) and 12 weeks (D_1_-F_3_) of post-surgery. A_1-3_ and D_1-3_: CTR group, B_1-3_ and E_1-3_: BGC group, C_1-3_ and F_1-3_: Cu-BGC group. (G_1_-L_3_) H&E staining at 8 weeks (G_1_-I_3_) and 12 weeks (J_1_-L_3_) of post-surgery. G_1-3_ and J_1-3_: CTR group, H_1-3_ and K_1-3_: BGC group, I_1-3_ and L_1-3_: Cu-BGC group. Van Gieson staining showed that Cu-BGC group possessed a certain amount of hyaline cartilage-like tissue, while CTR group exhibited a disordered structure in the defect region and BGC group possessed a disorder and discontinuous hyaline cartilage-like tissue at 8 weeks. Cu-BGC group was filled with a considerable amount of hyaline cartilage-like tissue, and exhibited well-integrated and orderly continuous structure between cartilage and neo-bone as compared to the CTR group and BGC group at 12 weeks. H&E staining indicated that the defect regions of BGC and Cu-BGC groups were filled with a great amount of bony tissue at 12 weeks, and that of CTR group possessed less and incomplete bony tissue.

In order to study the stimulating effect of Cu-BGC scaffolds on regeneration of osteochondral tissue, histological analysis, including H&E (Hematoxylin-eosin) and Van Gieson staining, was processed (Figure 10). These results showed that the neo-bone tissues and hyaline-like cartilage tissues in three experimental groups at week 12 were much more than that at week 8. H&E staining revealed that the osteochondral defects still exist large vacancy in CTR (blank control) group at week 8 and 12 (Figure 10G_1-3_ and J_1-3_), while the defects in BGC and Cu-BGC groups were covered with newly formed bone tissue at 12 weeks (Figure 10K_1-3_ and L_1-3_). Van Gieson staining showed that a mixture of new bone and fibrous tissues filled the defect region of CTR group at 8 and 12 weeks, and a vacancy was observed in the defect region (Figure 10A_1-3_ and D_1-3_). At 8 weeks, BGC and Cu-BGC groups contained a considerable quantity of hyaline-like cartilage tissues, and the hyaline-like cartilage tissue of Cu-BGC group was relatively continuous as compared with that of BGC group (Figure 10B_1_-C_3_). At 12 weeks, the defect region was completely covered with neo-bone and hyaline cartilage tissues in BGC and Cu-BGC groups. Neo-cartilage tissue in Cu-BGC group was orderly continuous, while the newly tissue in BGC group was disorder. In addition, a smooth and well-integrated interface between newly-formed cartilage and subchondral bone was found in Cu-BGC group, and the tide mark closely resemble to the surrounding natural tissue (Figure 10E_1_-F_3_). Compared to CTR and BGC groups, Cu-BGC group displayed a higher ICRS (International Cartilage Repair Society) score at 12weeks implantation (Figure S8).

## Discussions

In our study, one of important results is that the ionic extracts from Cu-BGC considerably improved the proliferation and maturation of chondrocytes as compared with BGC without Cu. Cu-BGC extracts significantly enhanced the gene expressions of COL II, ACAN and SOX-9. Furthermore, expressions of COL II protein and NCAD protein were also enhanced. As revealed in previous reports, chondrocytes were in a hypoxia environment and HIF-1α was important for the response of chondrocytes in hypoxia 28-30. Activation of HIF signaling pathway stimulated the expression of SOX-9, and the expression level of downstream COL II and ACAN was elevated 31, 32.

In this study, the expression level of HIF-1α gene distinctly enhanced after co-culturing with the ionic products from Cu-BGC, which followed by the increased SOX-9, COL II and ACAN. Therefore, the stimulating effects of Cu-BGC scaffolds on cartilage regeneration may refer to the activation of HIF signaling pathway via Cu^2+^ ions. In order to verify this, the relative genes and HIF-1α gene in chondrocytes incubated with different Cu^2+^ concentrations were further investigated. The results exhibited that the gene expression of HIF-1α was obviously enhanced as compared to CTR group. The expression of SOX-9 in chondrocytes was improved, and the increasing trend is consistent with HIF-1α within the corresponding concentration range. Besides, COL II and ACAN expression in chondrocytes treated with different Cu^2+^ ions concentrations were enhanced. Therefore, Cu^2+^ ions themselves released from Cu-BGC scaffolds play an important role in promoting the differentiation of chondrocytes and the repair of cartilage by activating the HIF pathway.

Interestingly, Cu-BGC ionic extracts have the capability to shift macrophages to an anti-inflammation M2 phenotype rather than pro-inflammation M1 phenotype. Macrophages are important immune cells, which have two extreme phenotypes, and the activation of macrophages affects the immune response 33, 34. The M1 phenotype of macrophages is pro-inflammatory, and could release the cytokines to facilitate immune response, while the M2 phenotype of macrophages could inhibit inflammation 35. Our results showed that the expression of pro-inflammatory cytokines (TNF-α and IL-18) in macrophages treated with Cu-BGC extracts was inhibited, while the anti-inflammatory cytokine (IL-10) was enhanced as compared with BGC group and CTR group. TNF-α is a major pro-inflammation cytokine, which involved in the catabolism of cartilage in the osteoarthritis 8, 36. Previously, TNF-α inhibited the synthesis of type II collagen and ACAN, and promoted the release of MMP-3 and MMP-13 37, 38. There is evidence showed that the inhibition of TNF-α decreased catabolism 8. On the other hand, interleukin-10 was regulated together with pro-inflammation cytokines, while IL-10 negatively regulates the pro-inflammation cytokines to protect the cartilage from immune damage 39.

Our previous publication showed that Cu-containing mesoporous silica nanospheres could stimulate macrophages to M1 phenotype. It is suggested that copper is not the only factor affecting the polarization of macrophages, and the nano-sized microstructure and particle size of nanospheres uptaken by macrophages may be one of important factors to influence their polarization 40. Another previous publication showed that the expression of M1 pro-inflammatory factor on macrophages after cultured with Cu-containing mesoporous bioactive glasses extracts was elevated, and the concentration of Cu^2+^ ions released from Cu-MBG (Cu-containing mesoporous bioactive glasses) was around 28.3 ppm 29, 41. However, according to our results, the effective concentration of Cu^2+^ ions released from Cu-BGC extracts was within the range of 0.5-16 ppm, which was lower than that of Cu-containing mesoporous bioactive glasses, thus we got a different result. The typical M1 phenotype macrophage surface marker iNOS in macrophage was inhibited, while the M2 phenotype macrophage surface marker CD206 was significantly increased in Cu-BGC group. Our results indicated that the M1 phenotype of macrophage was suppressed, while M2 phenotype was promoted. To verify the stimulating effect of Cu^2+^ ions on macrophages, the macrophages (RAW264.7) cells were cultured with different concentrations of Cu^2+^ ions. Results demonstrated that the expressions of the relative inflammatory cytokine and cell surface markers in macrophages cultured with different concentrations of Cu^2+^ ions obtained a similar result with Cu-BGC extracts. Therefore, Cu^2+^ ions could promote the inflammatory response via its cytotoxic effect at a higher concentration (28.3 ppm), and induce macrophages to shift to anti-inflammatory phenotype within a concentration range of 0.5-16 ppm. Based on the above studies, it is rational to speculate that the Cu^2+^ ions released from Cu-BGC scaffolds may selectively inhibit the catabolism in chondrocytes and promote the M2 phenotype of macrophage to reduce the damage of cartilage tissue in osteoarthritis within certain concentration ranges. Hence, Cu^2+^ ions for inducing proper anti-inflammatory phenotype are concentration dependent.

Cartilage damage and destruction of subchondral bone manifest in different stages of osteoarthritis 28. In the clinical, the treatment of osteoarthritis, including autologous chondrocytes (or bone marrow mesenchymal stem cell) transplantation as well as microfracture, has positive effects on cartilage regeneration 1, 42-45. However, there were few pieces of evidence indicated that these treatments alleviated the development of osteoarthritis 46. It is reported that pro-inflammation cytokines are not specific to the immune cells, and chondrocytes also secreted pro-inflammation cytokines under the inflammatory stimuli, and further accelerate cartilage damage through the action of reactive oxygen species 3, 8, 36, 47. Moreover, the inflammatory environment with pro-inflammation cytokines has been proved to limit the regeneration of cartilage. Therefore, it is vital for the regeneration of cartilage *via* activating the immune response in osteoarthritis. Furthermore, the anti-TNF and IL-10 agents were applied in clinical osteoarthritis therapy 8, 48. In addition, the effects of inflammation in osteoarthritis have been ignored in most of the previous studies for the regeneration of osteochondral tissue 49. In our study, Cu-BGC not only promoted the maturation of chondrocytes, but also regulated the shift of macrophages to anti-inflammatory phenotype. Furthermore, the *in vivo* study displayed a positive regeneration effect on osteochondral tissue. According to the results of the* in vitro* and *in vivo* studies, Cu-BGC not only activating the immune responses in osteochondral tissues, but also improving the repair of cartilage and subchondral bone tissues, indicating its promising application in preventing the development of osteoarthritis.

## Conclusion

Cu-BGC scaffolds were produced via a 3D-printed technology with stably released of Cu^2+^ ions. Cu-containing ionic products from Cu-BCG stimulate the maturation of chondrocytes. Cu-BGC scaffolds improved the regeneration of cartilage and facilitated the construction of osteochondral interface. The underlying mechanism might relate to Cu^2+^ ions facilitating the proliferation and maturation of chondrocytes through activating HIF pathway then further promoting the anti-inflammatory M2 phenotype and elevating the secretion of anti-inflammatory cytokines in macrophages to reduce the damage of cartilage tissue. The study demonstrates that Cu-BGC possesses the capability to regulate the immune responses *in vitro* and facilitate the regeneration of osteochondral tissue, which represents a feasible strategy to prevent the development of osteoarthritis associated with osteochondral defects.

## Supplementary Material

Supplementary figures and table.

## Figures and Tables

**Table 1:** The ionic concentration of Cu, Ca, Si and P in Cu-BGC extracts

Ionic Con. (mg/L)	0 (CTR)	Powders extracts concentrations (ppm)							
		BGC				Cu-BGC			
		0.781	3.125	12.5	25	0.781	3.125	12.5	25
Cu	0.00±0.00	0.00±0.00	0.00±0.00	0.00±0.00	0.00±0.00	0.50±0.01	2.00±0.05	8.00±0.19	16.02±0.39
Ca	69.31±3.03	69.73±3.05	71.00±3.09	76.05±3.27	82.79±3.52	69.21±2.11	68.91±3.01	67.72±3.00	66.13±2.89
Si	0.24±0.09	0.75±0.07	2.29±0.02	8.45±0.22	16.67±0.52	0.70±0.07	2.09±0.03	7.65±0.16	15.07±0.41
P	26.06±1.69	25.96±1.68	25.67±1.66	24.50±1.58	22.94±1.47	25.98±1.69	25.75±1.67	24.85±1.60	23.64±1.51

## Abbreviations

BGC: bioactive glass ceramics
Ca(NO_3_)_2_·4H_2_O: calcium nitrate tetrahydrate
Cu(NO_3_)_2_·3H_2_O: copper nitrate trihydrate
CCK-8: cell counting kit-8
CLSM: confocal laser scanning microscope
COL II: type II collagen protein
CTR: control group
Cu: copper
Cu-BGC: copper-incorporated bioactive glass ceramics
DAPI: 4',6-diamidino-2-phenylindole
DMEM: dulbecco's modified eagle's medium low-glucose
F-127: poloxamer
FITC: fluorescein isothiocyanate phalloidin
HE: hematoxylin-eosin
HMDS: hexamethyldisilazane
HNO_3_: nitric acid
IL-10: interleukin-10
IL-18: interleukin-18
iNOS: inducible nitric oxide synthase
Micro-CT: microcomputed tomography
NCAD: N-cadherin
RT-qPCR: quantitative real-time reverse transcriptase polymerase chain reaction
SD: standard deviation
SEM: scanning electron microscope
TEOS: Tetraethyl orthosilicate
TNF-α: tumor necrosis factor-α
