# Supplementary material for: Copper-incorporated bioactive glass-ceramics inducing anti-inflammatory phenotype and regeneration of cartilage/bone interface
Source: Theranostics. 2019 Aug 14;9(21):6300–13. doi: 10.7150/thno.36120 (PMC6735521; doi:10.7150/thno.36120)
Supplement: Supplementary file 1 — Supplementary figures and table. [file thnov09p6300s1.pdf]

Supporting information

for "Copper-incorporated bioactive glass-ceramics inducing anti-inflammatory phenotype and regeneration of cartilage/bone interface"

Rongcai Lin[1#], Cuijun Deng[2, 3#], Xuxiang Li[1], Yaqin Liu[2], Meng Zhang[2], Chen Qin[2], Qingqiang Yao[1,*], Liming Wang[1,*], Chengtie Wu[2*]

1. Department of Orthopaedics, Nanjing First Hospital, Nanjing Medical University. Nanjing 210006, P.R.China

2. State Key Laboratory of High Performance Ceramics and Superfine Microstructure, Shanghai Institute of Ceramics, Chinese Academy of Sciences. Shanghai 200050, P.R.China

3. Shanghai East Hospital, the Institute for Biomedical Engineering & Nano Science, Tongji University School of Medicine, 150 Jimo Road, Shanghai 200092, P.R.China

* **Corresponding Author**

Email: limingwang99@yahoo.com

Email: yaoqingqiang@126.com

Email: chengtiewu@mail.sic.ac.cn

#Rongcai Lin and Cuijun Deng are co-first authors.

Table S1. The primer sequences used for RT-qPCR analysis

Gene	Forward primer	Reverse primer
GAPDH (rabbit)	5-TCACCATCTTCCAGGAGCGA	5-CACAATGCCGAAGTGGTCGT
COL II	5-AACACTGCCAACGTCCAGAT	5-CTGCAGCACGGTATAGGTGA
ACAN	5-AGGTCGTGGTGAAAGGTGTTG	5-GTAGGTTCTCACGCCAGGGA
SOX-9	5-GGTGCTCAAGGGCTACGACT	5-GGGTGGTCTTTCTTGTGCTG
HIF-1α	5-GCCACCACTGACGATTAAAACC	5-GGTGATGTTGTGGCACTAGC
GAPDH (mouse)	5-TGACCACAGTCCATGCCATC	5-GACGGACACATTGGGGGTAG
TNF-α	5-CTGAACTTCGGGGTGATCGG	5-GGCTTGTCACTCGAATTTTGAGA
IL-18	5-TGGCCGACTTCACTGTACAAC	5-TGGGGTTCACTGGCACTTTG
IL-10	5-GAGAAGCATGGCCCAGAAATC	5-GAGAAATCGATGACAGCGCC
iNOS	5-CAGAAGTGCAAAGTCTCAGACAT	5-GTCATCTTGTATTGTTGGGCT
CD206	5-AGACGAAATCCCTGCTACTG	5-CACCCATTCGAAGGCATTC

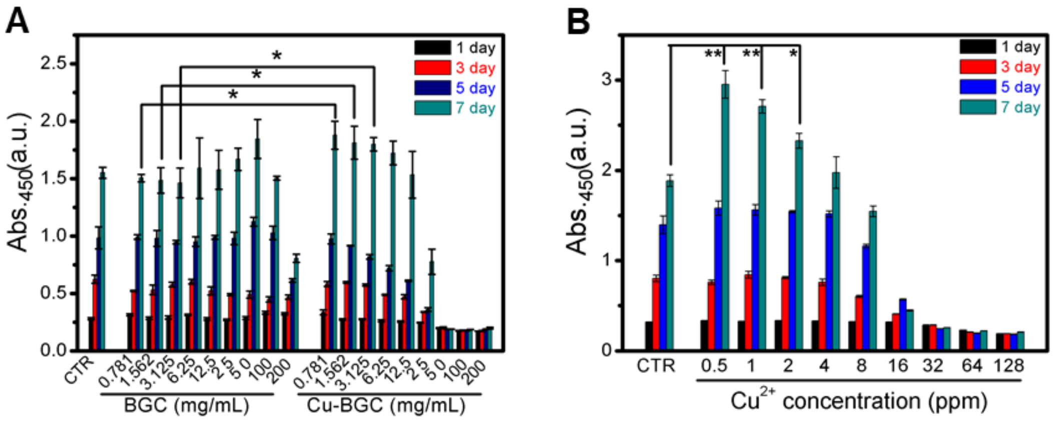

Figure S1. The proliferation of chondrocytes treated with BGC, Cu-BGC ionic extracts and different concentrations of Cu^{2+} ions. As compared with CTR group, the proliferation of chondrocytes treated with different concentrations of Cu-BGC extracts (A) and Cu^{2+} ions (B) was improved within a certain concentration range, respectively. (n=6, *p<0.05, **p<0.01, ***p<0.001)

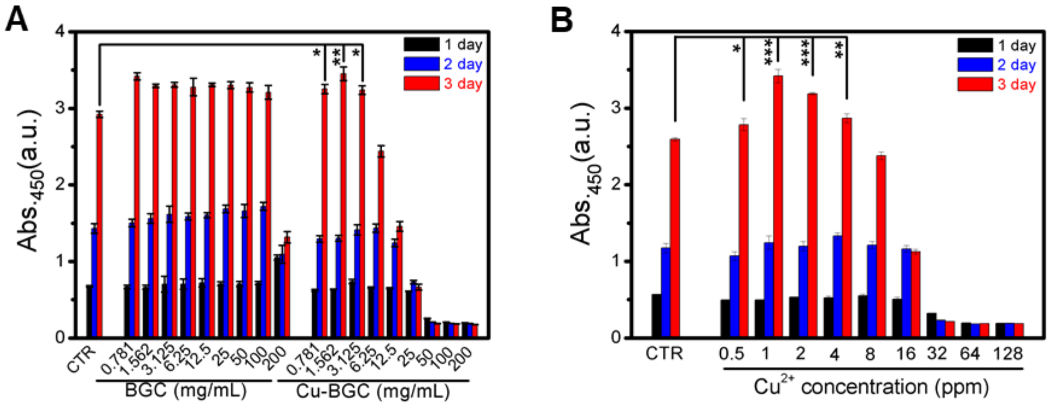

Figure S2. The proliferation of macrophages. The proliferation of macrophages cultured with the ionic products of Cu-BGC (A) and different concentrations of Cu^{2+} ions (B) was enhanced within certain concentrations range, respectively. (n=6, *p<0.05, **p<0.01, ***p<0.001)

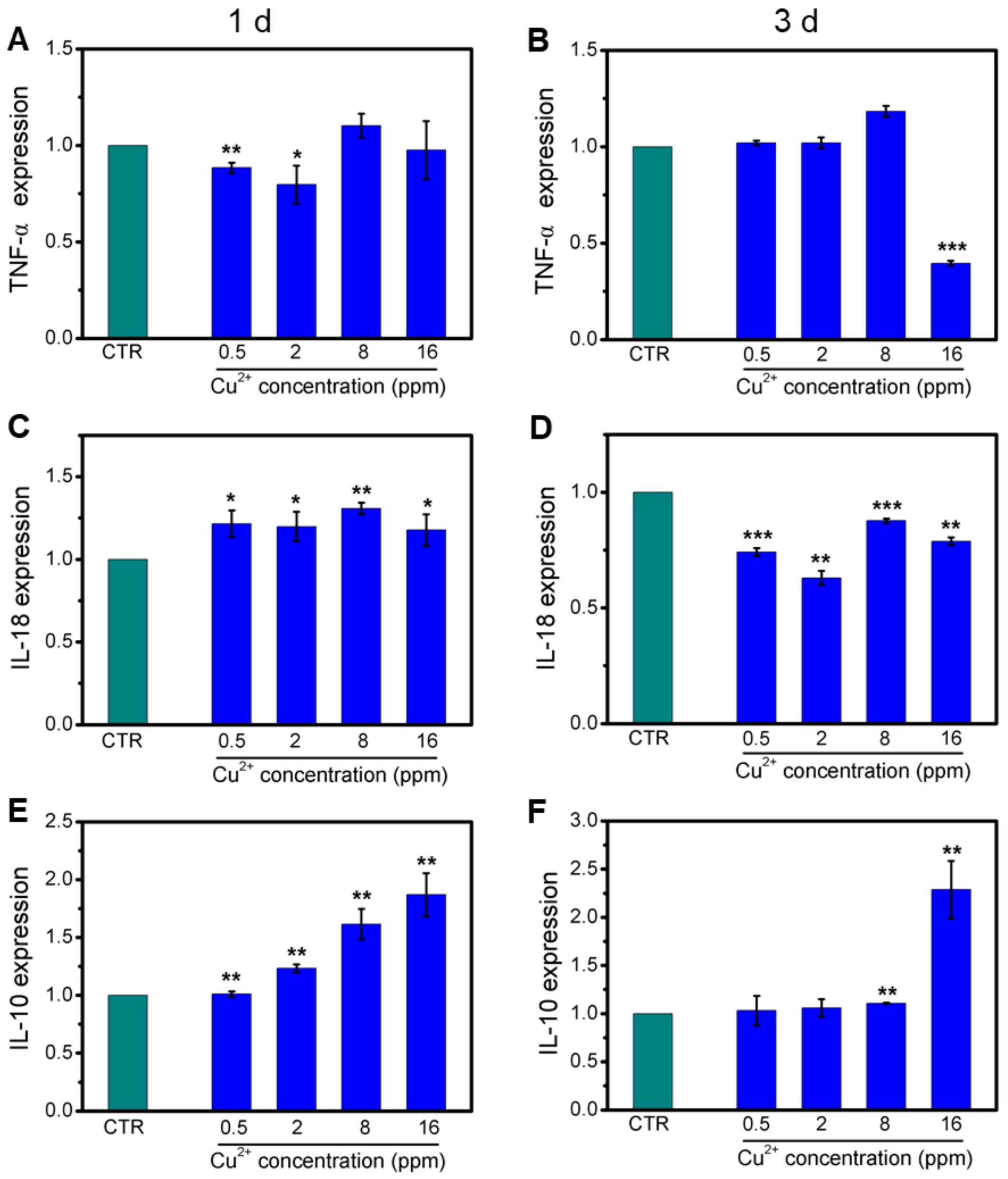

Figure S3. The inflammatory cytokine expression in macrophages stimulated by different concentrations of Cu^{2+} ions. The expression of pro-inflammatory cytokine (TNF-α: A, B; IL-18: C, D) was inhibited, and the anti-inflammatory cytokine (IL-10: E, F) was enhanced after treating with different concentrations of Cu^{2+} ions for 3 days. The relative gene amount of CTR group was set as 1. (n=3, *p<0.05, **p<0.01, ***p<0.001)

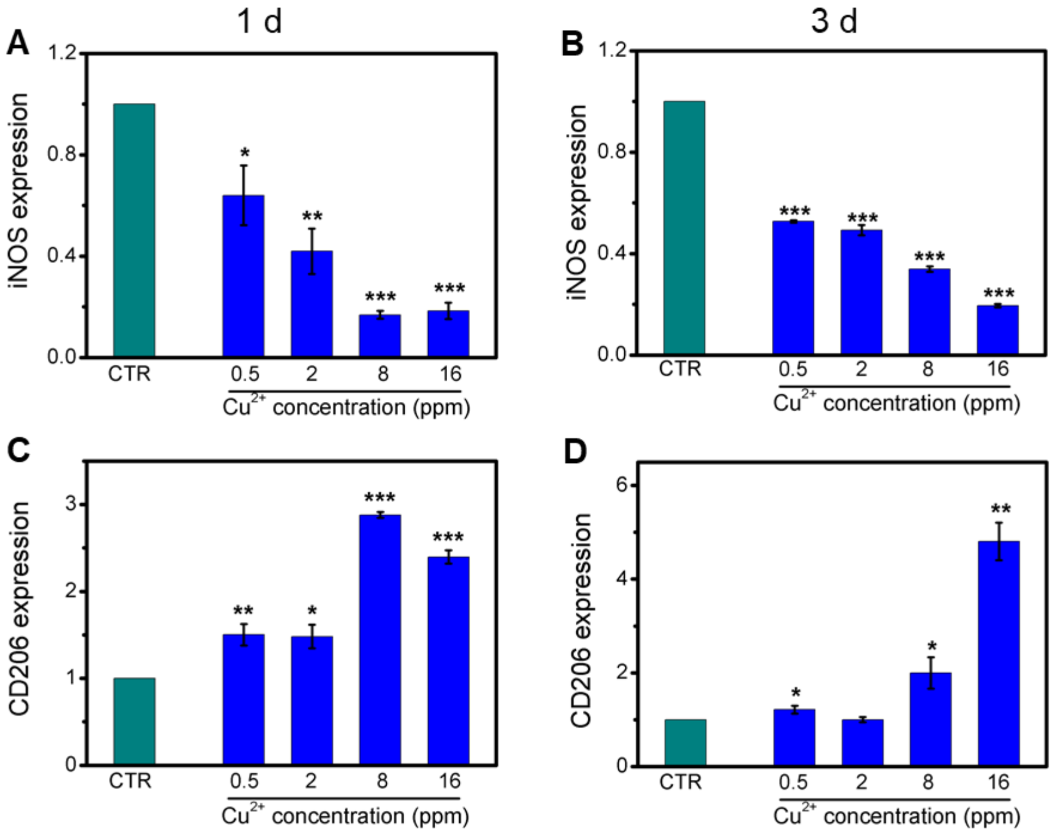

Figure S4. The expression of macrophage surface markers in macrophage after treating with different concentrations of Cu^{2+} ions at day 1 and 3. (A, B) M1 marker: iNOS; (C, D) M2 marker: CD206. The macrophage phenotype was changed to an anti-inflammatory M2 phenotype after treating with different concentrations of Cu^{2+} ions. The relative gene amount of CTR group was set as 1. (n=3, *p<0.05, **p<0.01, ***p<0.001)

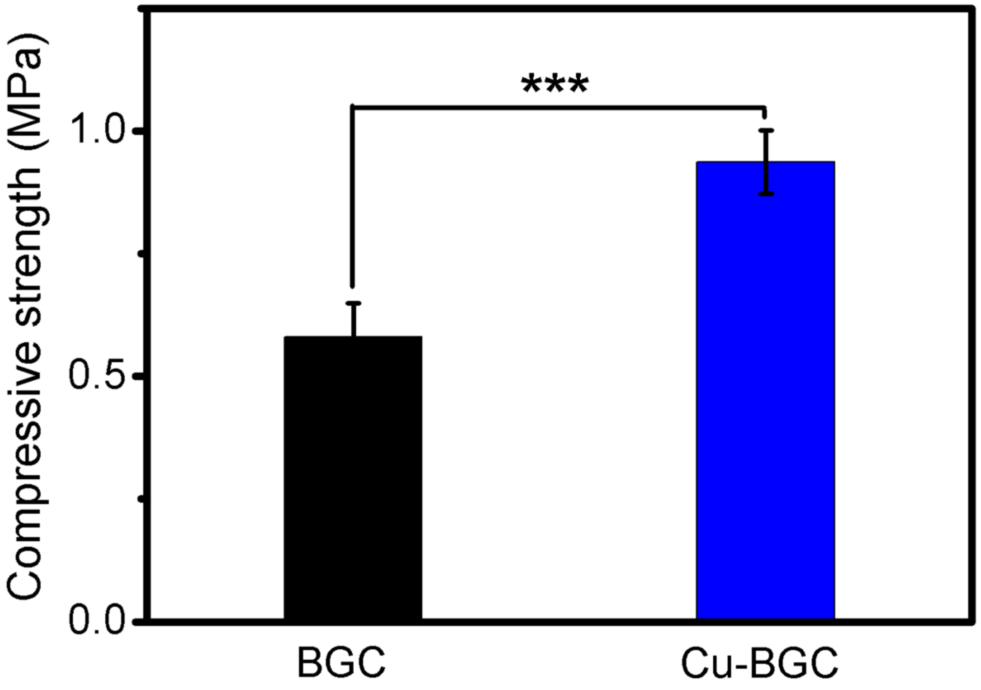

Figure S5. The compressive strength of BGC and Cu-BGC scaffolds. (A) BGC scaffold, (B) Cu-BGC scaffold. The results indicated that Cu-BGC scaffolds possess a higher compressive strength as compared with BGC scaffolds. (n=6, *p<0.05, **p<0.01, ***p<0.001)

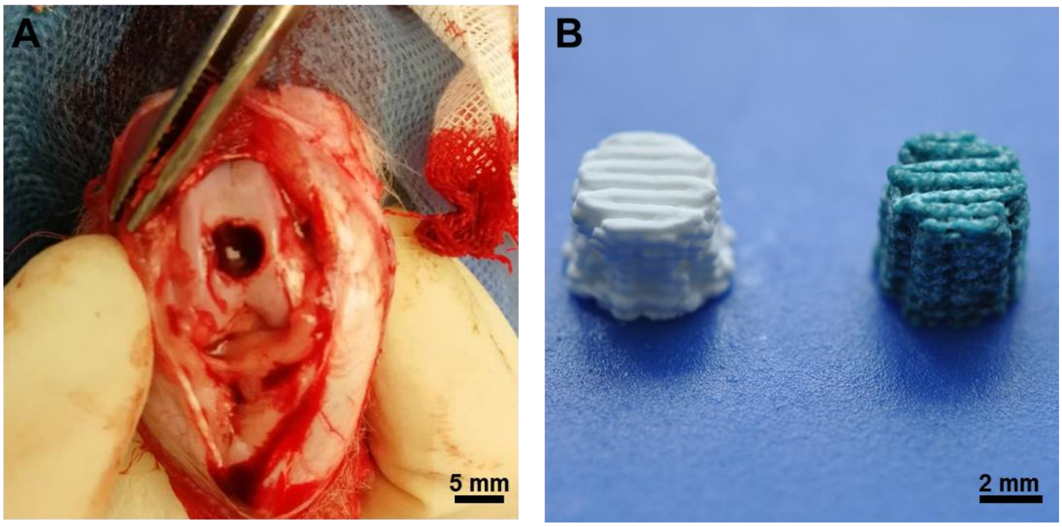

Figure S6. The digital photographs of the defect and the scaffolds during the surgery. (A) The osteochondral defect was 5 mm high and 5 mm in diameter. (B) The size of scaffolds which were implanted into the defects was 5 mm high and 5 mm in diameter.

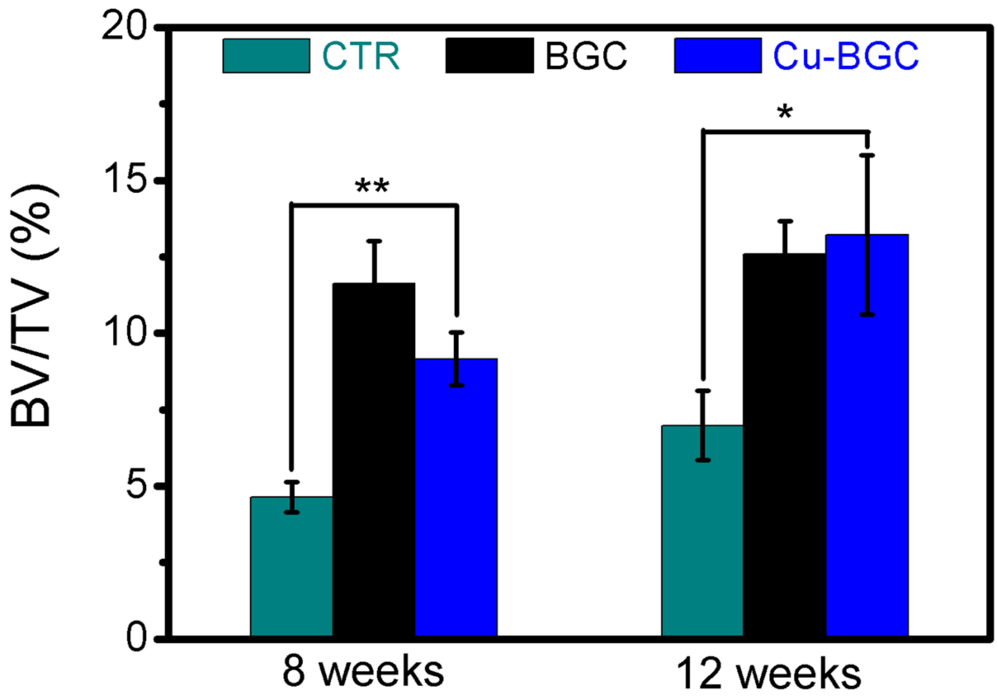

Figure S7. Micro-CT imaging analysis (BV/TV) of the defects at 8 and 12 weeks. Micro-CT analysis of defect space showed that it was greater level of bone formation in Cu-BGC group as compared to CTR group. (n=6, *p<0.05, **p<0.01, ***p<0.001)

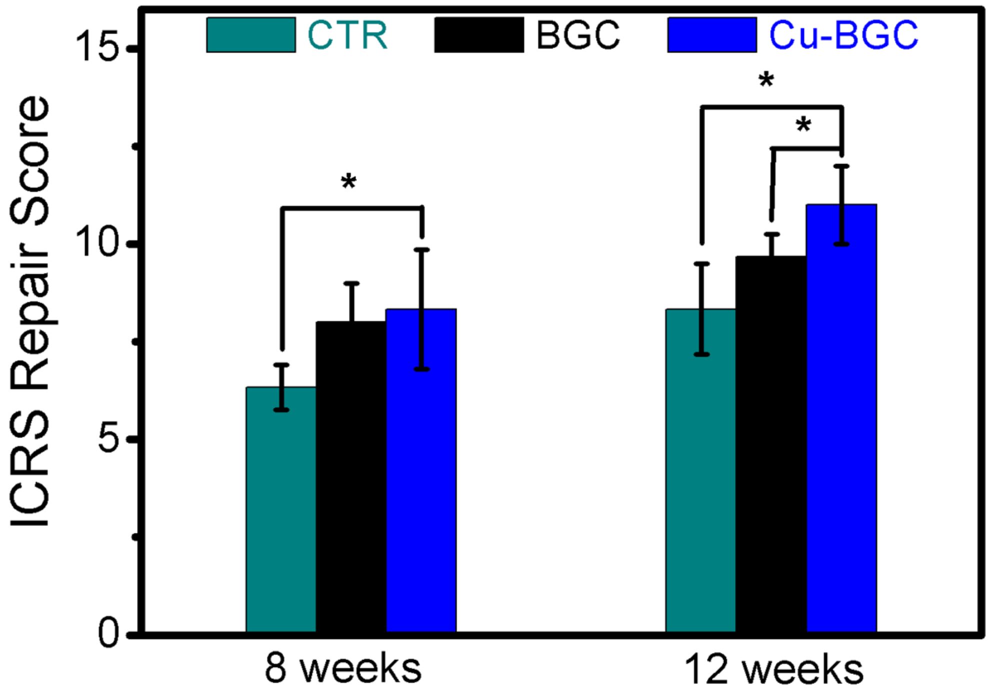

Figure S8. The ICRS scores of knee samples at 8 and 12 weeks of postsurgery. Blinded quantity of ICRS scores for CTR, BGC and Cu-BGC groups by three investigators, the results showed that the ICRS score of Cu-BGC group was enhanced as compared to CTR and BGC groups at 12 weeks. (n=6, *p<0.05, **p<0.01, ***p<0.001)
